# Transcriptome analysis of interactions between silkworm and cytoplasmic polyhedrosis virus

**DOI:** 10.1038/srep24894

**Published:** 2016-04-27

**Authors:** Liang Jiang, Zhengwen Peng, Youbing Guo, Tingcai Cheng, Huizhen Guo, Qiang Sun, Chunlin Huang, Ping Zhao, Qingyou Xia

**Affiliations:** 1 State Key Laboratory of Silkworm Genome Biology, Southwest University, Chongqing 400715, P. R. China

## Abstract

*Bombyx mori* cytoplasmic polyhedrosis virus (BmCPV) specifically infects silkworm midgut (MG) and multiplication occurs mainly in posterior midgut (PM). In this study, MG and fat body (FB) were extracted at 0, 3, 24, and 72 h after BmCPV infection. The total sequence reads of each sample were more than 1510000, and the mapping ratio exceeded 95.3%. Upregulated transcripts increased in MG during the infection process. Gene ontology (GO) categories showed that antioxidants were all upregulated in FB but not in MG. BGI001299, BGI014434, BGI012068, and BGI009201 were MG-specific genes with transmembrane transport function, the expression of which were induced by BmCPV. BGI001299, BGI014434, and BGI012068 expressed in entire MG and may be involved in BmCPV invasion. BGI009201 expressed only in PM and may be necessary for BmCPV proliferation. BmPGRP-S2 and BGI012452 (a putative serine protease) were induced by BmCPV and may be involved in immune defense against BmCPV. The expression level of BmCPV S1, S2, S3, S6, and S7 was high and there was no expression of S9 in MG 72 h, implying that the expression time of structural protein coding genes is earlier. These results provide insights into the mechanism of BmCPV infection and host defense.

Viral diseases cause serious threats to human health and also in the breeding of animals and plants. The study of host–virus interactions is an important interest in the antivirus field. The silkworm *Bombyx mori* is an economically important insect for silk production, and sericulture is a principal source of income for farmers in many developing countries such as China, India and so on. However, viruses, such as *B. mori* nucleopolyhedrovirus (BmNPV), *B. mori* cytoplasmic polyhedrosis virus (BmCPV), and *B. mori* densovirus (BmDNV), cause huge economic losses^1^. Recently, with the completion of the silkworm genome project^2^,^3^ and the development of transgenic technology^4^, antivirus research has been rapidly promoted in the silkworm^1^,^4^. Based on numerous studies of silkworm and BmNPV interactions, multiple antivirus strategies have been performed to generate anti-BmNPV silkworms^1^. However, there are few reports about silkworm and BmCPV interactions, and this hinders research towards a BmCPV antivirus.

BmCPV is a double-stranded RNA (dsRNA) virus, which is a member of the CPV subfamily that belongs to the genus *Cypovirus* of family *Reoviridae*^5^. The genome of BmCPV contains ten discrete equimolar dsRNA segments, in which S1, S2, S3, S4, S6, and S7 encode virus structural proteins, while S5, S8, S9, and S10 encode nonstructural proteins^6^. There is a protein capsid and nucleic acid but no envelope in the BmCPV particle^7^. CPV particles are occluded within polyhedral bodies with a random spatial distribution^8^, of which the 3.88 Å three-dimensional structure was analyzed using single-particle cryo-electron microscopy^9^. BmCPV infects the silkworm larvae *per os*, which specifically invades the midgut (MG) columnar cells and causes white wrinkles in the posterior midgut (PM)^7^,^10^.

The MG is an important organ for virus invasion and host defense in silkworm. Some MG-specific genes play key roles in the interaction of the virus and host^1^,^11^. BmDNV-2 only infects the silkworm MG but causes a fatal disease. The receptor of BmDNV-2 was assumed to be +^*nsd−2*^, which encodes a transmembrane protein that is expressed only in the MG. A 6 kb deletion in the open reading frame of +^*nsd−2*^ resulted in resistance to BmDNV-2^12^. Some proteins with strong antiviral activity against BmNPV were expressed specifically in MG, including Bmlipase-1, BmSP-2, BmNOX, and RFPs^1^,^11^. Overexpression of *Bmlipase-1* can enhance the anti-BmNPV capacity of the transgenic silkworm^13^,^14^. However, no specific MG genes that participate in BmCPV infection or host defense have been identified.

With the advent of next-generation sequencing technologies, there has been a rapid development in genome-wide studies of host and virus interactions. Recently, several studies on the transcriptional response of silkworm against BmCPV challenge have been reported; these focused on differentially expressed genes^15^,^16^,^17^,^18^ and miRNAs^19^, and host RNAi-related genes^20^. The primary issue in BmCPV study is that why this virus only infects the MG of silkworm. We think that comparison of the invaded MG and non-invaded tissue (such as fat body, FB) is a viable strategy for solve this problem. In this study, the MG and FB of silkworms were extracted over time after oral infection with BmCPV for analysis by RNA-seq. We focused on analysis of the factors that may affect BmCPV specifically infecting MG and the viral expression pattern, which has not received much attention in previous research.

## Results

### Transcriptome sequencing and genome-guided assembly

RNA libraries from the MG and FB after BmCPV challenge were sequenced in our lab using Illumina paired-end sequencing technology. The total reads of each sample were more than 15100000 (Table S1). After removing adapters, low-quality sequences and ribosomal RNA, the clean reads were mapped to the reference database of silkworm using TopHat. The mapping ratio of each sample exceeded 95.3% (Table S1). The clean reads were assembled into transcripts using Cufflinks^21^. The level of all transcripts of MG 0 h, MG 3 h, MG 24 h, MG 72 h, FB 0 h, FB 3 h, FB 24 h, and FB 72 h are shown in Supplementary Material-2. Figure S1 shows the dispersion plot of all transcripts of eight tissues samples.

### Differentially expressed transcripts analysis and gene ontology analysis

**Figure 1:**
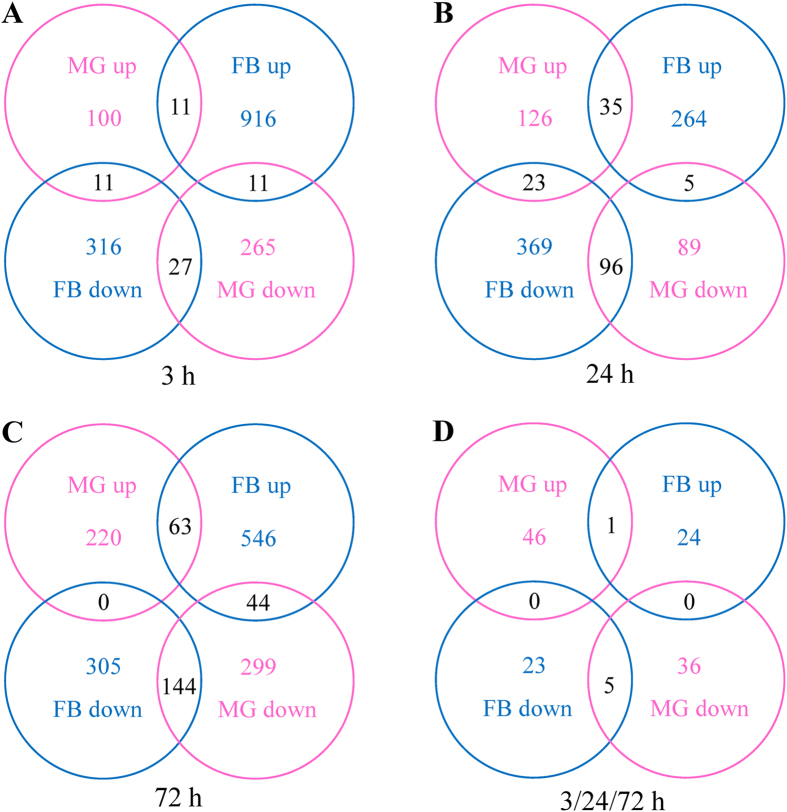
Differentially expressed transcripts statistics. Transcripts expression levels of MG 3 h, MG 24 h, and MG 72 h were compared to MG 0 h, respectively; while FB 3h, FB 24 h, and FB 72 h were compared to FB 0 h, respectively. The up- and down-expressed transcripts were identified by expression ratios higher than 2.0 and lower than 0.5. (**A**) Differentially expressed transcripts of MG 3 h were compared to FB 3 h. (**B**) MG 24 h was compared to FB 24 h. (**C**) MG 72 h was compared to FB 72 h. (**D**) Common differentially expressed transcripts of MG 3 h, 24 h, and 72 h were compared to that of FB.

The transcripts expressed levels of MG 3 h, MG 24 h, and MG 72 h were compared to MG 0 h, respectively; while the transcripts expressed levels of FB 3 h, FB 24 h, and FB 72 h were compared to FB 0 h, respectively. The up- and down-expressed transcripts were abstracted from Cuffdiff results when statistical significance of each observed changes in expression is yes. Then, the differentially expressed transcripts of MG 3 h were compared to FB 3 h (Fig. 1A), MG 24 h were compared to FB 24 h (Fig. 1B), and MG 72 h were compared to FB 72 h (Fig. 1C). There were 100, 126, and 220 transcripts upregulated only in 3 h, 24 h, and 72 h of MG (Fig. 1), respectively, which indicated that the infection process increased upregulated transcripts in the MG. The up and down transcripts of FB were more than that of MG at each time point (Fig. 1), suggesting that BmCPV affects multiple organizations of silkworm even though this virus only infects MG. One transcript was co-upregulated and five transcripts were co-downregulated in all the time points of the two tissues (Fig. 1D); the levels of the six transcripts are shown in Table S2.

**Figure 2:**
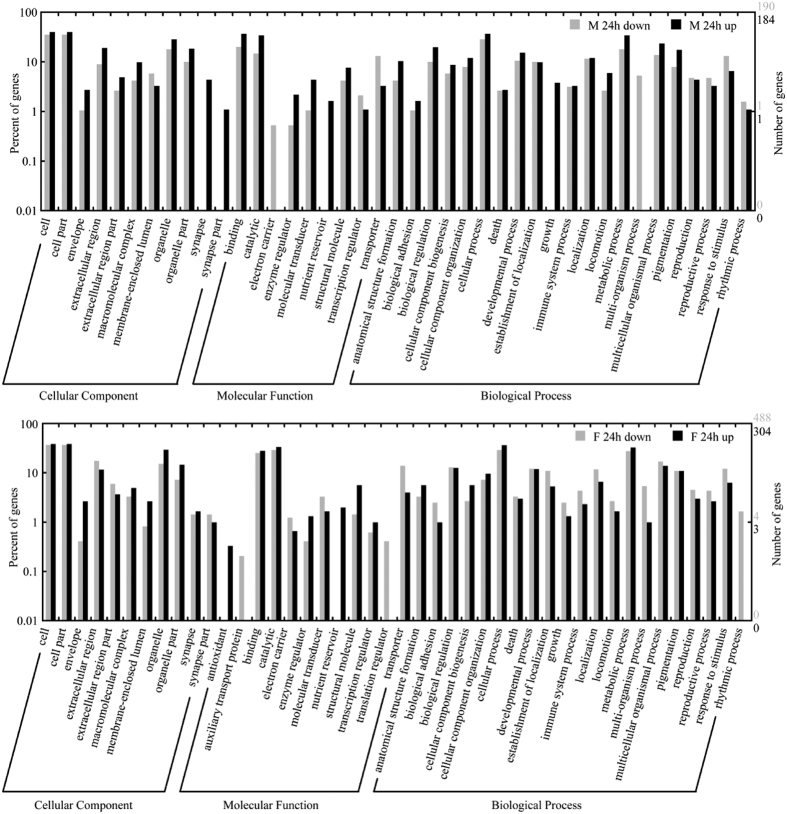
GO annotation of differentially expressed transcripts of MG 24 h and FB 24 h. M 24 h down and M 24 h up represent the downregulated and upregulated transcripts of MG 24 h. F 24 h down and F 24 h up typify the downregulated and upregulated transcripts of FB 24 h.

The differentially expressed transcripts of MG 3 h, MG 24 h, MG 72 h, FB 3 h, FB 24 h, and FB 72 h were analyzed for gene ontology (GO) categories, which are divided into cellular component, molecular function, and biological process domains and widely used to classify gene function. In each sample, cell, cell part, binding, catalytic, cellular process, and metabolic process were dominant GO classifications (Fig. 2 and Fig. S2). Rhythmic process was upregulated in MG 3 h, while it was downregulated in FB 3 h. Antioxidant, enzyme regulator, metallochaperone, and nutrient reservoir were all upregulated in FB 3 h (Fig. S2A). Synapse, synapse part, nutrient reservoir, and growth were all upregulated while electron carrier and multi-organism process were all downregulated in MG 24 h. Antioxidant and nutrient reservoir were all upregulated while auxiliary transport protein, translation regulator, and rhythmic process were all downregulated in FB 24 h (Fig. 2). Antioxidant was all downregulated in MG 72 h. Antioxidant, auxiliary transport protein, chemoattractant, metallochaperone, nutrient reservoir, and translation regulator were all upregulated in FB 72 h (Fig. S2B). The KEGG ontology assignments of differentially expressed transcripts were also performed, the results were shown in Supplementary Material-3.

### Analysis of antioxidant-related genes

**Figure 3:**
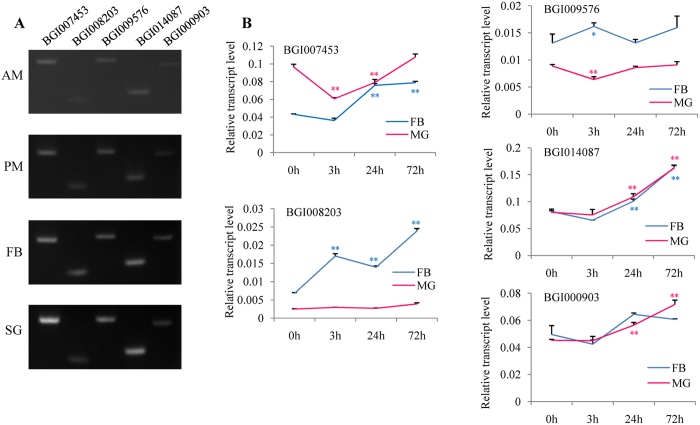
Analysis of genes related to antioxidant. (**A**) RT-PCR analysis of BGI007453, BGI008203, BGI009576, BGI014087, and BGI000903 with different tissues of day 3 of the fifth instar. AM, anterior midgut; PM, posterior midgut; FB, fat body; SG, silk gland. (**B**) qPCR analysis with the cDNA of MG 0 h, MG 3 h, MG 24 h, MG 72 h, FB 0 h, FB 3 h, FB 24 h, and FB 72 h. Bars, standard deviations. Statistically significant differences: *P < 0.05, **P < 0.01.

The antioxidant was upregulated in FB but not in MG (Fig. 2 and Fig. S2), suggesting that the antioxidant-related genes may prevent BmCPV from infecting FB. Further bioinformatics analysis showed that there were 19 antioxidant-related transcripts, which belong to the BGI007453, BGI008203, BGI009576, BGI014087, and BGI000903 genes (Table S3). RT-PCR results showed that these five genes were all expressed in AM, PM, FB, and SG (Fig. 3A). qPCR analysis revealed that BGI007453 and BGI008203 were upregulated in FB but not MG, BGI014087 was induced in FB and MG, and BGI000903 was upregulated in MG after BmCPV challenge (Fig. 3B). Some transcripts were considered not expressed and not in the GO categories because of their low level; this resulted in the qPCR results being not exactly the same as the GO classification.

### Analysis of induced MG-specific genes with transmembrane transport function

**Figure 4:**
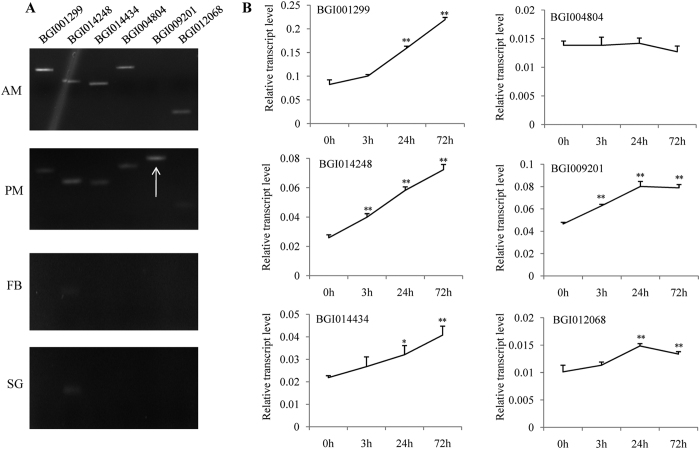
Analysis of induced MG-specific genes with transmembrane transport function. BGI001299, BGI014248, BGI014434, BGI004804, BGI009201, and BGI012068 were detected. (**A**) RT-PCR with AM, PM, FB, and SG. (**B**) qPCR with MG 0 h, MG 3 h, MG 24 h, and MG 72 h. Bars, standard deviations. Statistically significant differences: *P < 0.05, **P < 0.01.

BmCPV specifically invades MG and the viral proliferation was mainly in the PM^7^,^10^. It presumed that certain MG-specific genes facilitate the BmCPV infection. There were 216 MG-specific genes in the silkworm^11^,^22^. Bioinformatics analysis showed that six MG-specific genes with transmembrane transport function were induced by BmCPV, including BGI001299, BGI014248, BGI014434, BGI004804, BGI009201, and BGI012068 (Table S4). RT-PCR indicated that BGI001299, BGI014434, BGI004804, and BGI012068 were specifically expressed in entire MG while BGI009201 was expressed only in PM (Fig. 4A). qPCR revealed that BGI001299, BGI014434, BGI009201, and BGI012068 were induced in MG by BmCPV (Fig. 4B and Fig. S3).

### Analysis of induced MG-specific genes related to host defense

**Figure 5:**
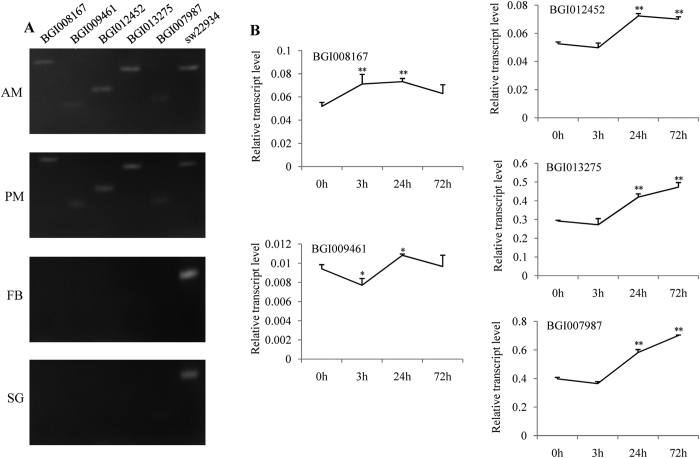
Analysis of induced MG-specific genes related to host defense. BGI008167, BGI009461, BGI012452, BGI013275, and BGI007987 were tested. TIF-4A was the internal control. (**A**) RT-PCR. (**B**) qPCR using BmCPV-infected MG. Bars, standard deviations. Statistically significant differences: *P < 0.05, **P < 0.01.

It was presumed that some MG-specific genes were involved in antivirus defense against BmCPV. BGI008167, BGI009461, BGI012452, BGI013275, and BGI007987 were filtered out, because these five genes are related to immune defense and induced by BmCPV challenge (Table S5). Bioinformatics analysis showed that BGI008167 and BGI012452 were putative serine proteases (SP), BGI009461 was a putative TNF receptor-associated factor 1, BGI013275 was a putative Zinc carboxypeptidase A 1, and BGI007987 was peptidoglycan recognition protein S2 (BmPGRP-S2). RT-PCR showed that BGI008167, BGI009461, BGI012452, and BGI013275 specifically expressed in the entire MG, BmPGRP-S2 was mainly expressed in MG and weakly expressed in SG (Fig. 5A). qPCR indicated that BGI012452, BGI013275, and BmPGRP-S2 were induced by BmCPV in MG 24 h and MG 72 h, BGI008167 was induced in MG 3 h and MG 24 h (Fig. 5B and Fig. S3).

### Analysis of MG-specific BmAPNs

**Figure 6:**
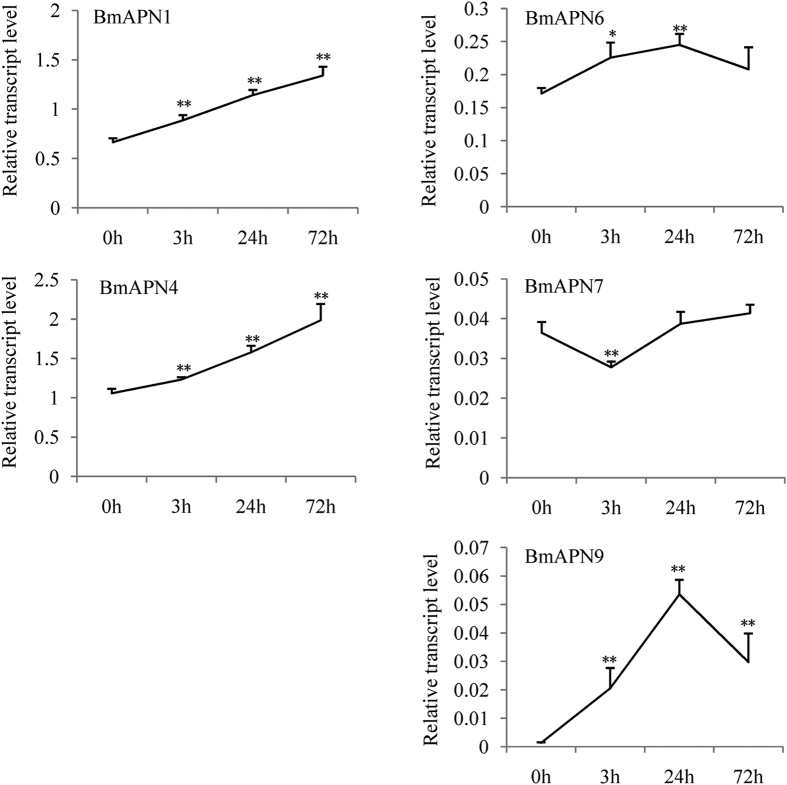
Analysis of MG-specific BmAPNs. BmAPN1, BmAPN4, BmAPN6, BmAPN7, and BmAPN9 were investigated in BmCPV-infected MG. Bars, standard deviations. Statistically significant differences: *P < 0.05, **P < 0.01.

Aminopeptidases N enzymes (APNs) are important immune genes that are well known as receptors of *Bacillus thuringiensis* (*Bt*) Cry toxin^23^. Some transcripts of BmAPNs were induced by BmCPV (data not shown). BmAPN1, BmAPN4, BmAPN6, BmAPN7, and BmAPN9 were specifically expressed in MG^24^. qPCR showed that BmAPN1, BmAPN4, and BmAPN9 were induced by BmCPV in MG 3 h, MG 24 h, and MG 72 h, BmAPN6 was upregulated in MG 3 h and MG 24 h, while BmAPN7 was downregulated in MG 3 h (Fig. 6). These results suggest that certain BmAPNs may be involved in the interaction of silkworm and BmCPV. The primers of BmAPNs were referred from the literature^24^, and the other primers used in this study were shown in Table S6.

### Actin involved in the interaction of BmCPV and silkworm

**Figure 7:**
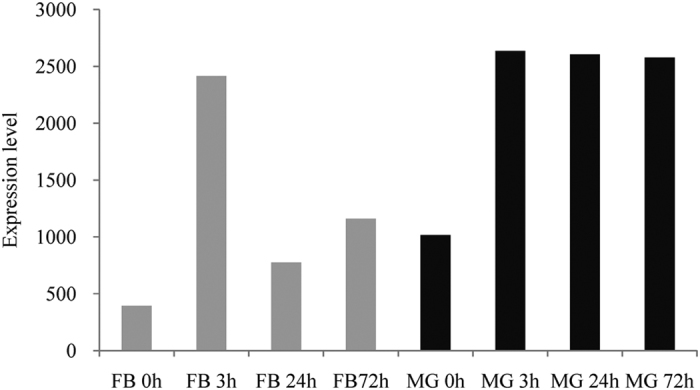
The expression level of actin.

Actin was induced by NPV and necessary for NPV infection in hosts^25^,^26^,^27^,^28^,^29^,^30^. Our results showed that it was also induced by BmCPV infection, in especially sustained high level in the MG. The level was 393.6, 2415.3, 773.9, 1159.4, 1015.6, 2634.9, 2604.0, and 2577.6 in FB 0 h, FB 3 h, FB 24 h, FB 72 h, MG 0 h, MG 3 h, MG 24 h, and MG 72 h, respectively (Fig. 7). These results suggested that actin is involved in the interaction of BmCPV and silkworm. The expression of clathrin in MG was also affected by BmCPV challenge (data not shown).

### Analysis of the expression pattern of BmCPV genes

**Figure 8:**
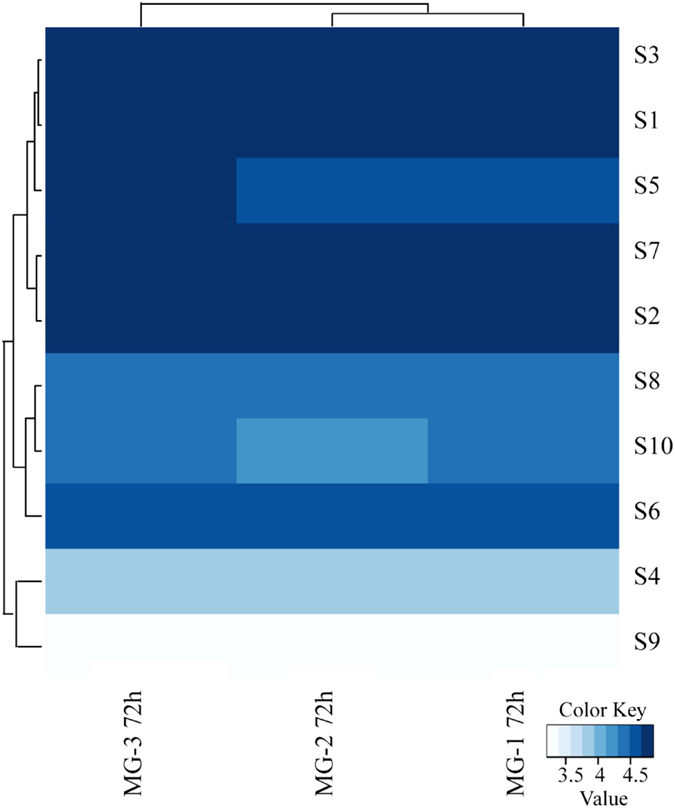
The expression level of BmCPV genes in MG 72 h. MG-1 72h, MG-2 72h, and MG-3 72h are triplicate sample repeats of MG 72 h.

The expression of BmNPV genes has a temporal pattern with four phases^31^. However, there is no report about the expression pattern of BmCPV genes. Our results showed that there were no viral genes expressed in FB 0 h, FB 3 h, FB 24 h, FB 72 h, MG 0 h, MG 3 h, and MG 24 h. S1, S2, S3, S4, S5, S6, S7, S8, and S10 but not S9 was expressed in MG 72 h, and the level of S1, S2, S3, S6, and S7 was very high (Fig. 8). Further RT-PCR analysis also confirmed that there was almost no viral gene expression in MG 24 h and MG 48 h (Fig. S4). These results suggested that the expression of BmCPV genes followed certain chronological events with S9 being the latest.

## Discussion

BmCPV is one of the primary pathogens of silkworm. However, the mechanisms of BmCPV infection and host defense are unknown, as a result no fundamental strategies have been established to control BmCPV in sericulture. In this study, we analyzed the interaction of silkworm and BmCPV in genome-wide via transcriptome, bioinformatics analysis, RT-PCR, and qPCR.

BmCPV only infects the MG of silkworm^7^,^10^. However, the expression of many genes was affected in MG and FB after BmCPV challenge (Fig. 1). Two reasons should be considered: (a) BmCPV regulates gene expression in FB via host signaling pathways to facilitate its multiplication. (b) Silkworm actively regulates the genes of multiple tissues by itself to copy with the virus infection. It is an interesting result that TCONS00010332, TCONS00010483, TCONS00042090, TCONS00054269, and TCONS00057208 were sustained downregulated while TCONS00055534 was sustained upregulated in MG and FB after BmCPV infection (Table S2), suggesting that the six transcripts may play important roles in BmCPV multiplication or host defense. Their functions are unclear and need further research. GO categories of differentially expressed transcripts showed that rhythmic process was upregulated in MG 3 h but downregulated in FB 3 h and FB 24 h (Fig. 2 and Fig. S2). Gao *et al.*^15^ found that rhythmic process was upregulated in BmCPV-infected 4008 silkworm. These results suggested that rhythmic process-related genes may be involved in the interaction between silkworm and BmCPV. The nutrient reservoir related genes were all upregulated in MG 24 h, FB 3 h, FB 24 h, and FB 72 h (Fig. 2 and Fig. S2), implying that the antivirus defense of the host would consume a large amount of energy.

The reasons for BmCPV specifically infecting MG may be: (a) The receptors of BmCPV are MG-specific genes, which are critical for virus invasion. There is no envelope out of nucleocapsid in BmCPV^7^ and BmDNV^32^, suggesting the two virions could not invade MG cells by membrane fusion. The receptor of BmDNV-2 is a transmembrane protein^12^. We presume that BmCPV invades MG cells by utilizing certain transmembrane proteins. Bioinformatics analysis showed that BGI001299 has an Aa-trans domain, BGI014434 contains a transmembrane region and MFS-1 domain, and BGI012068 has a transmembrane region and C2 domain, suggesting that they have transmembrane transport function. The three genes are specifically expressed in entire MG and induced by BmCPV (Fig. 4), implying that they might be the receptors of BmCPV. We will knock out the three genes for further functional studies in the next experiments. A surprising result was that MG-specific BmAPN1, BmAPN4, BmAPN6, and BmAPN9 were upregulated after BmCPV infection (Fig. 6). APNs were well known as receptors of *Bt* toxin^23^. Loss and mutation of APN cause resistance of the host to *Bt* toxin^24^,^33^,^34^. Whether these BmAPNs are involved in BmCPV invasion requires further experiments. (b) Certain MG-specific genes are necessary for BmCPV proliferation. BmCPV causes white wrinkles only in PM^7^,^10^, revealing that the multiplication of BmCPV occurs mainly in PM. BGI009201 contains MFS-1 and sugar transporter domains, which was only expressed in PM and upregulated after BmCPV challenge (Fig. 4). To confirm if BGI009201 is necessary for BmCPV multiplication, in subsequent studies we plan to construct transgenic silkworm in which overexpression of BGI009201 is by AM-specific promoter P2^11^ and knock out BGI009201 by genome editing techniques. (c) Certain antivirus genes inhibit the BmCPV infection in other tissues. BGI007453 and BGI008203, antioxidant-related genes, are induced after BmCPV challenge in FB but not MG (Fig. 3). There is a close correlation between virus disease and oxidative stress^35^. The elevated oxidants induced by viral infection may promote viral pathogenesis and the regulation of viral proliferation^36^,^37^. In order to prevent reactive oxygen species-mediated harm caused by virus infection, antioxidants in the host cell react with elevated oxidants and superoxides and suppress virus multiplication^38^. It is presumed that BGI007453 and BGI008203 may have antivirus activity related to BmCPV not infecting FB.

Some candidate MG-specific antivirus genes against BmCPV were also identified in this study. BGI012452 and BGI013275 are MG-specific genes and induced by BmCPV (Fig. 5). BGI012452 is a putative SP. BmSP-2 is expressed in the entire MG of silkworm, which has a signal sequence and strong antivirus activity to BmNPV in MG juice^39^. Bioinformatics analysis indicated that BGI012452 contains a signal peptide, Peptidase S28, and Abhydrolase 6 domains, suggesting this protein could secrete into gut juice for antivirus against BmCPV. Overexpression of this gene using entire MG-specific prompter would be an available strategy for its function research. BGI013275 is a putative Zinc carboxypeptidase; predicted results show that it has the signal peptide, Propep M14, and Zn pept domains. It might be involved in the defense against BmCPV in gut juice. BmPGRP-S2 is primarily expressed in MG and induced by BmCPV (Fig. 5). Gao *et al.*^40^ showed that BmPGRP-S3 is presented in all of the tissues but highest in MG, and that this expression is induced by BmCPV. Their results and those of the current study suggest that BmPGRPs may play an important role in immune defense against BmCPV infection in silkworm.

The expression of actin and clathrin are induced after BmCPV challenge (Fig. 7), suggesting that the two genes participate in the interaction of silkworm and BmCPV. Actin is one of the cytoskeleton-associated proteins, which play an important role in BmNPV infection^27^,^28^. Actin is necessary for viral gene expression and facilitates released nucleocapsid transport into the nucleus^25^,^26^,^27^,^28^,^29^,^30^, of which the expression level is stimulated by BmNPV P95 protein^41^. The results of the literatures^25^,^26^,^27^,^28^,^29^,^30^ and our current study reveal that BmCPV and BmNPV utilize some of the same host factors for their multiplication and actin is necessary for the intracellular transportation of the two viruses. Clathrin is an evolutionarily highly conserved protein which mediates endocytosis and regulates the entry of pathogens^42^. The entry of hepatitis C virus (HCV) can be inhibited by arbidol, which affects clathrin-mediated endocytosis (CME) by impeding dynamin-2-induced membrane scission^43^. Silibinin also inhibits HCV in the early steps of infection by hindering clathrin-dependent trafficking^44^. It is interesting that compounds pitstops can block endocytic ligand association with the terminal domain of clathrin, this could be a potential virus inhibitor^42^. BmCPV entry is possibly by means of viropexis^7^, in this process clathrin may play an important role. It was presumed that a small molecule compound target CME may inhibit the infection of BmCPV.

Viral genes were expressed in MG 72 h but not in MG 24 h and MG 48 h (Fig. S4). The expression level of S1, S2, S3, S6, and S7 is higher than that of S5, S8, S9, and S10 in MG 72 h (Fig. 8). S1, S2, S3, S6, and S7 encode the virus structural proteins while S5, S8, S9, and S10 encode the nonstructural proteins^6^. We presume that the expression pattern of BmCPV genes would be that structural protein coding genes are expressed earlier than those of nonstructural protein coding genes. Therefore, when structural proteins coding genes are highly expressed in the early stage of infection, the level of nonstructural protein coding genes are lower.

In conclusion, the RNA-seq of MG and FB after BmCPV challenge were performed to analyze silkworm and BmCPV interactions. We found that BGI001299, BGI014434, BGI009201, and BGI012068 may be key genes affecting how BmCPV specifically infects MG, BGI012452 and BmPGRP-S2 are involved in antivirus defense against BmCPV, and the expression patterns of BmCPV genes reveal structural protein coding genes that are expressed earlier than nonstructural protein coding genes. The data and analysis presented here provide insights into the mechanism of BmCPV infection and host defense and a basis for future antivirus studies.

## Materials and Methods

### Tissue sample collection

Silkworm strain Dazao was orally infected by BmCPV with 10^6^ OB/larva at the newly exuviated fifth instar. The time that the infection process ended was set as time point 0 h. The MG and FB were collected at 0 h, 3 h, 24 h, and 72 h post-infection. Thirty treated larvae were used for tissue collection at each time point. Each tissue contained triplicate sample repeats, and every repeat was from ten treated larvae. The 24 samples were named as MG 0 h-1, MG 0 h-2, MG 0 h-3, MG 3 h-1, MG 3 h-2, MG 3 h-3, MG 24 h-1, MG 24 h-2, MG 24 h-3, MG 72 h-1, MG 72 h-2, MG 72 h-3, FB 0 h-1, FB 0 h-2, FB 0 h-3, FB 3 h-1, FB 3 h-2, FB 3 h-3, FB 24 h-1, FB 24 h-2, FB 24 h-3, FB 72 h-1, FB 72 h-2, and FB 72 h-3.

### RNA extraction and RNA-seq

Total RNA of each sample was extracted using an SV Total RNA Isolation System (Promega Z3100) and treated with Rnase-free Dnase I (Promega). The quality and quantity of 24 RNA samples were analyzed, RNA libraries were constructed and sequenced as described^45^,^46^. Raw sequencing data were generated using an Illumina HiSeq 2000 system and have been deposited in the NCBI Short Read Archive.

### RNA-seq data analysis

To generate clean reads, raw reads were filtered of polyA tails using fqtrim (v0.93), aligned to the tRNA and rRNA libraries of silkworm with Bowtie2^47^ (v2.2.3) for removing noncoding RNA, and low-quality reads removed with Trimmomatic^48^ (v0.32). The qualities of raw and clean RNA-seq reads were analyzed using FastQC (v0.11.1). Clean reads were mapped to the silkworm genome with TopHat (v2.0.12), and the genome sequences and annotation file were downloaded from SilkDB (http://www.silkdb.org/silkdb/doc/download.html). The aligned reads were then used to construct transcripts with Cufflinks (v2.1.1). Differential expression analysis was performed using cuffdiff which take advantage of the negative binomial distribution to calculate the P-value for determining the significance level^30^, and the results were visualized using CummeRbund (v2.0.0)^21^. GO analysis of differential expression transcripts was executed using WEGO online^49^. KEGG annotation of differential expression transcripts was performed using iPathCons^50^ standalone blastx with the KO terms databases of *B. mori* and *Drosophila melanogaster*. Clean reads were also mapped to the BmCPV genome with Bowtie (v2.2.3), and RSEM (v1.2.19) was employed to identity reads from BmCPV and calculate the expression of virus genes in MG 72 h.

### RT-PCR and qPCR analysis

Anterior midgut (AM), PM, FB, and silk gland (SG) of day 3 fifth instar larvae (Dazao) were dissected for RNA extraction. A total of 2 μg RNA after Dnase treatment was reverse-transcribed in a 25 μL reaction system using M-MLV reverse transcriptase (Promega)^13^. These cDNA were used for RT-PCR analysis; TIF-4 A was used as a control. The cDNA of MG 0 h, MG 3 h, MG 24 h, MG 72 h, FB 0 h, FB 3 h, FB 24 h, and FB 72 h were used for qPCR reactions, TIF-4 A was the internal control, and each detection was performed thrice^13^.

## Additional Information

**How to cite this article**: Jiang, L. *et al.* Transcriptome analysis of interactions between silkworm and cytoplasmic polyhedrosis virus. *Sci. Rep.* **6**, 24894; doi: 10.1038/srep24894 (2016).

## Supplementary Material

Supplementary Dataset 1

Supplementary Dataset 2

Supplementary Dataset 3
