# Supplementary material for: Transcriptome analysis of interactions between silkworm and cytoplasmic polyhedrosis virus
Source: Sci Rep. 2016 Apr 27;6:24894. doi: 10.1038/srep24894 (PMC4847007; doi:10.1038/srep24894)
Supplement: Supplementary Dataset 1 [file srep24894-s1.doc]

Transcriptome analysis of interactions between silkworm and cytoplasmic polyhedrosis virus

Liang Jiang ^†^, Zhengwen Peng ^†^, Youbing Guo ^†^, Tingcai Cheng, Huizhen Guo, Qiang Sun, Chunlin Huang, Ping Zhao, Qingyou Xia ^*^

*State Key Laboratory of Silkworm Genome Biology, Southwest University, Chongqing 400715, P. R. China*

†These authors contribute equally to this work.

*Correspondence author. Address: State Key Laboratory of Silkworm Genome Biology, Southwest University, Chongqing 400715, China

Tel: +86 23 68250099

Fax: +86 23 68251128

*E-mail address*: [xiaqy@swu.edu.cn](mailto:xiaqy@swu.edu.cn) (Q.Xia)


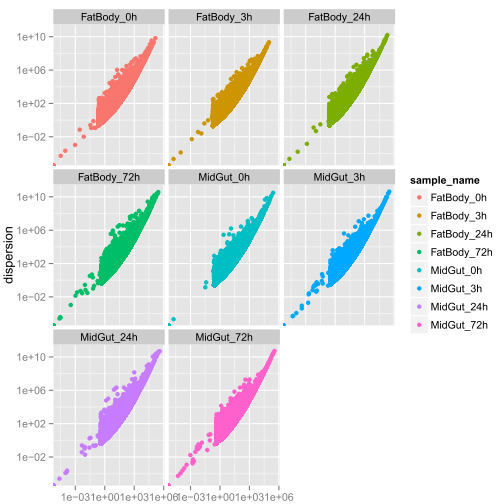


**Figure S1.** Count versus dispersion plot by condition for all transcripts.

**A**


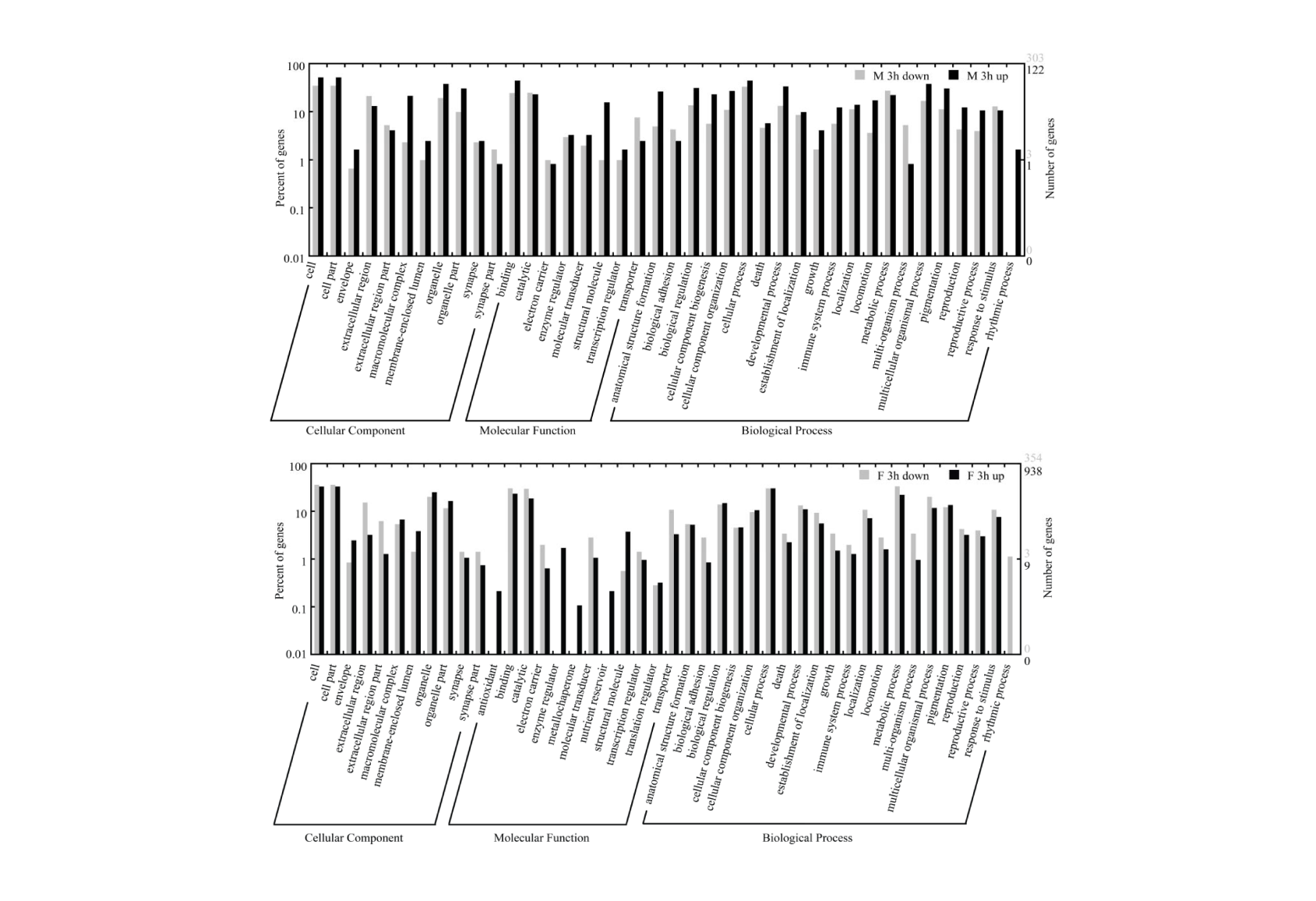


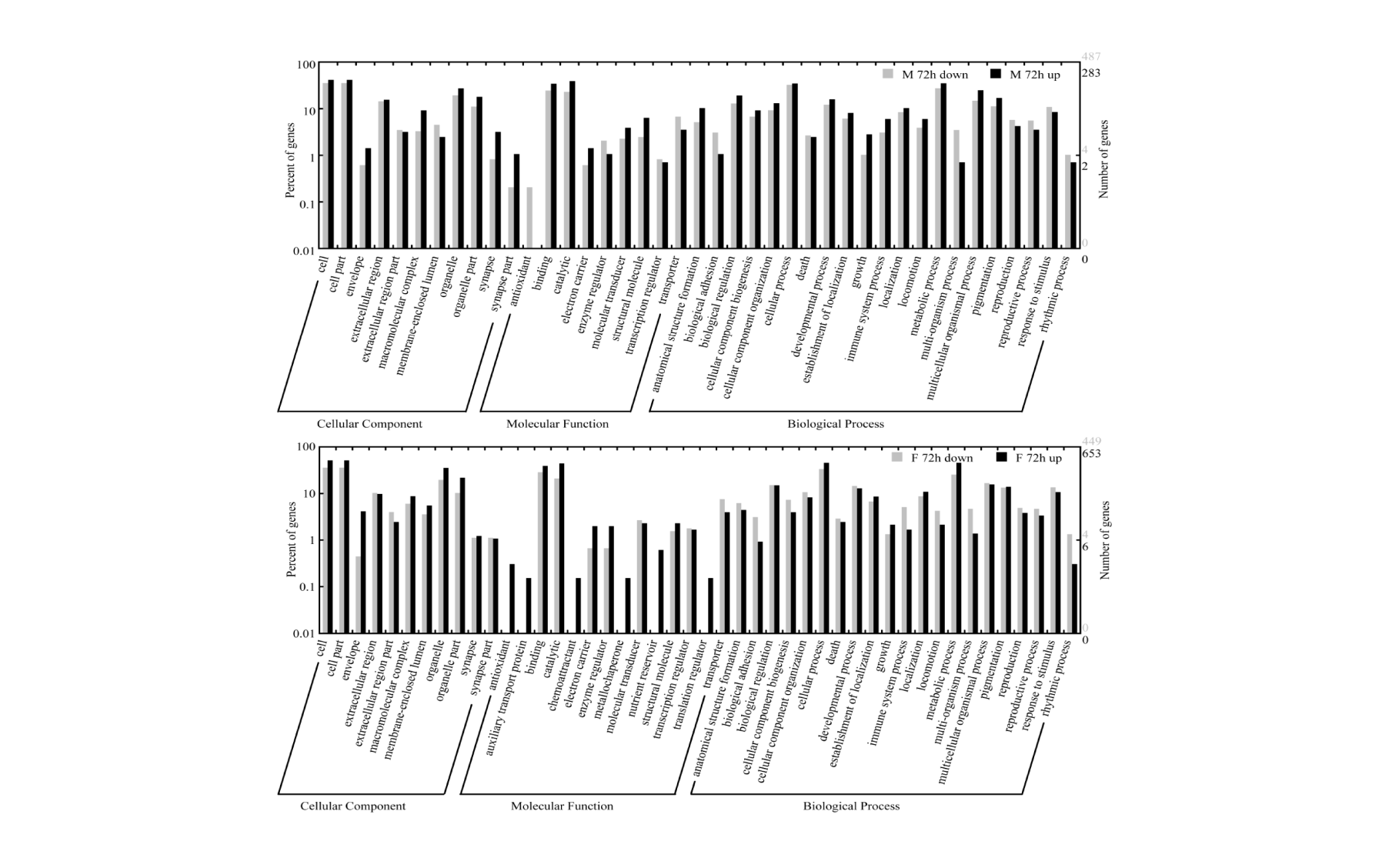


**B**

**Figure S2.** GO annotation of differentially expressed transcripts of 3h (A) and 72h (B).


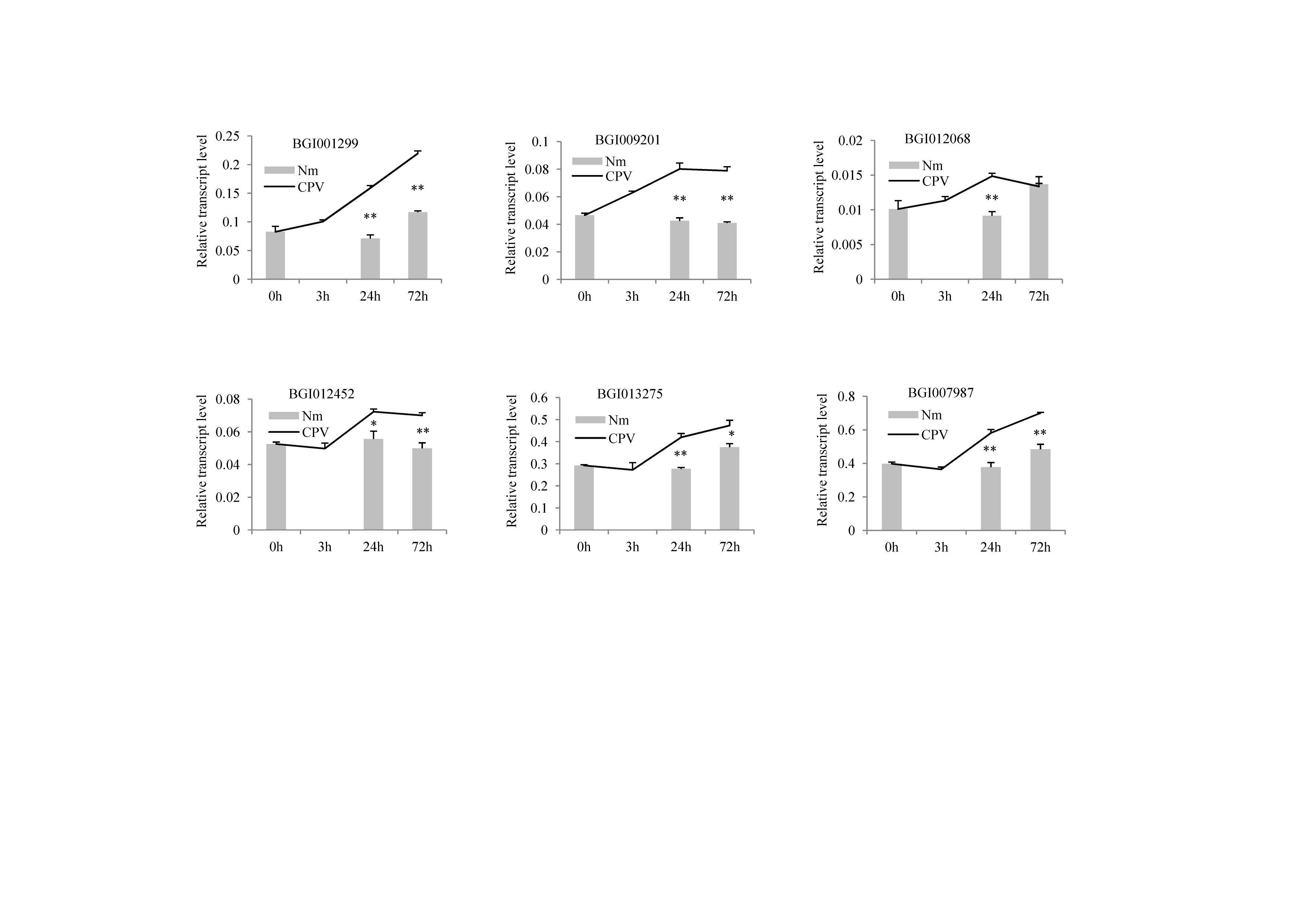


**Figure S3.** qPCR analysis of representative genes in different development stages. The BmCPV infected midgut (CPV) was collected at 0 h, 3 h, 24 h, and 72, while the noninfected midgut (Nm) was collected at 0 h, 24 h, and 72 h. These samples were used for analysis of representative genes (BGI001299, BGI009201, BGI012068, BGI012452, BGI013275, and BGI007987) by qPCR.

**
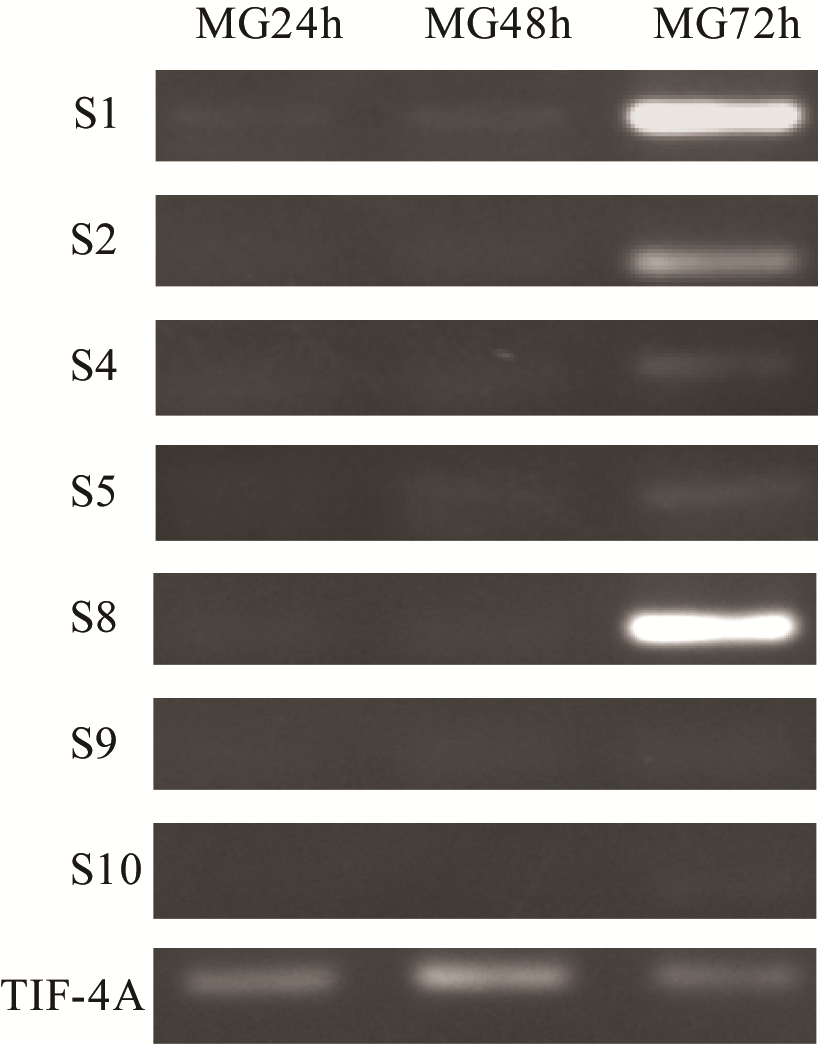
**

**Figure S4.** Analysis of the expression patterns of BmCPV genes by RT-PCR. The midgut was collected at 24 h, 48 h, and 72 h post-infection. BmCPV S1, S2, S4, S5, S8, S9, S10, and control TIF-4A were tested by RT-PCTR.

**Table S1.** Summary of reads

| Sample | Total Reads | Left input | Right input | Over Read Mapping Rate | Concordant Pair Alignment Rate |
| --- | --- | --- | --- | --- | --- |
| FB 0h-1 | 16422487 | 14226014 | 14226014 | 95.50% | 91.80% |
| FB 0h-2 | 17433767 | 15446555 | 15446555 | 95.50% | 92.10% |
| FB 0h-3 | 15678253 | 13789983 | 13789983 | 95.50% | 92.10% |
| FB 3h-1 | 17666624 | 14647390 | 14647390 | 95.80% | 92.30% |
| FB 3h-2 | 18607954 | 15708740 | 15708740 | 95.80% | 92.50% |
| FB 3h-3 | 18019389 | 14108465 | 14108465 | 95.70% | 92.10% |
| FB 24h-1 | 16757173 | 15585744 | 15585744 | 95.30% | 91.80% |
| FB 24h-2 | 17949391 | 16468569 | 16468569 | 95.40% | 92.00% |
| FB 24h-3 | 16121202 | 15814535 | 15814535 | 95.40% | 92.00% |
| FB 72h-1 | 15452958 | 13524882 | 13524882 | 96.00% | 92.60% |
| FB 72h-2 | 15347088 | 13731237 | 13731237 | 96.30% | 93.60% |
| FB 72h-3 | 16259118 | 14539010 | 14539010 | 96.30% | 93.50% |
| MG 0h-1 | 16099800 | 13737438 | 13737438 | 95.60% | 91.80% |
| MG 0h-2 | 16413551 | 14215936 | 14215936 | 95.50% | 91.80% |
| MG 0h-3 | 17898119 | 15222712 | 15222712 | 95.70% | 91.90% |
| MG 3h-1 | 15486476 | 14849679 | 14849679 | 95.60% | 91.80% |
| MG 3h-2 | 15131972 | 13553669 | 13553669 | 95.90% | 92.50% |
| MG 3h-3 | 16752707 | 14371333 | 14371333 | 95.70% | 91.90% |
| MG 24h-1 | 17138542 | 13186470 | 13186470 | 95.50% | 91.60% |
| MG 24h-2 | 15492095 | 12919002 | 12919002 | 95.30% | 91.30% |
| MG 24h-3 | 16586273 | 14409779 | 14409779 | 95.30% | 91.50% |
| MG 72h-1 | 15636485 | 13653109 | 13653109 | 95.60% | 91.70% |
| MG 72h-2 | 16746586 | 14563269 | 14563269 | 95.40% | 91.50% |
| MG 72h-3 | 16559743 | 14347350 | 14347350 | 95.80% | 91.90% |

**Table S2.** The levels of co-changed transcripts of MG and FB in all the time points.

| Isoform ID | FB 0h | FB 3h | FB 24h | FB72h | MG 0h | MG 3h | MG 24h | MG 72h |
| --- | --- | --- | --- | --- | --- | --- | --- | --- |
| TCONS_00055534 | 1.21 | 7.14 | 3.48 | 5.56 | 5.34 | 20.42 | 21.75 | 23.51 |
| TCONS_00010332 | 21.19 | 8.44 | 6.25 | 3.70 | 14.91 | 7.80 | 5.22 | 4.58 |
| TCONS_00010483 | 15.51 | 5.90 | 4.18 | 1.45 | 7.55 | 2.46 | 2.15 | 1.73 |
| TCONS_00042090 | 87.81 | 39.75 | 16.88 | 9.29 | 39.34 | 15.36 | 11.41 | 8.48 |
| TCONS_00054269 | 50.59 | 12.58 | 3.48 | 1.68 | 44.81 | 22.84 | 5.80 | 3.51 |
| TCONS_00057208 | 181.63 | 54.55 | 19.31 | 1.21 | 41.72 | 0.56 | 0.27 | 0.32 |

**Table S3.** The levels of transcripts related to antioxidant.

| Gene ID | Isoform ID | FB 0h | FB 3h | FB 24h | FB72h | MG 0h | MG 3h | MG 24h | MG 72h |
| --- | --- | --- | --- | --- | --- | --- | --- | --- | --- |
| BGIBMGA007453 | TCONS_00028832 | 0.29 | 18.15 | 1.26 | 0.00 | 0.01 | 0.05 | 0.00 | 0.29 |
| BGIBMGA007453 | TCONS_00028834 | 0.14 | 26.69 | 0.52 | 0.00 | 0.08 | 0.12 | 0.14 | 0.00 |
| BGIBMGA008203 | TCONS_00031682 | 0.26 | 1.18 | 1.05 | 4.78 | 0.26 | 0.24 | 0.09 | 0.12 |
| BGIBMGA008203 | TCONS_00031683 | 7.07 | 14.63 | 16.91 | 28.88 | 5.86 | 5.41 | 9.19 | 5.49 |
| BGIBMGA008203 | TCONS_00031684 | 0.32 | 1.71 | 1.32 | 1.19 | 0.25 | 0.27 | 0.52 | 0.53 |
| BGIBMGA008203 | TCONS_00031685 | 0.14 | 0.00 | 0.00 | 0.00 | 0.00 | 0.12 | 0.07 | 0.05 |
| BGIBMGA008203 | TCONS_00031686 | 0.00 | 0.51 | 0.00 | 0.36 | 0.28 | 1.31 | 0.42 | 0.88 |
| BGIBMGA009576 | TCONS_00037096 | 44.10 | 53.82 | 59.00 | 78.65 | 57.20 | 62.76 | 66.64 | 72.51 |
| BGIBMGA009576 | TCONS_00037097 | 35.25 | 32.71 | 33.50 | 59.24 | 25.94 | 39.07 | 47.84 | 53.13 |
| BGIBMGA014087 | TCONS_00053504 | 0.02 | 5.34 | 3.69 | 5.85 | 7.20 | 0.02 | 0.02 | 0.02 |
| BGIBMGA014087 | TCONS_00053505 | 100.75 | 64.29 | 170.58 | 278.48 | 112.92 | 95.62 | 137.29 | 127.07 |
| BGIBMGA000903 | TCONS_00004694 | 0.00 | 12.99 | 0.30 | 0.08 | 0.04 | 0.00 | 0.00 | 0.00 |
| BGIBMGA000903 | TCONS_00004699 | 25.78 | 10.19 | 35.59 | 35.67 | 40.02 | 39.90 | 35.24 | 32.06 |
| BGIBMGA000903 | TCONS_00004697 | 2.25 | 1.80 | 0.78 | 1.08 | 2.60 | 1.65 | 1.06 | 0.85 |
| BGIBMGA000903 | TCONS_00004701 | 24.67 | 12.81 | 31.90 | 42.39 | 38.40 | 33.10 | 30.80 | 36.27 |
| BGIBMGA000903 | TCONS_00004696 | 8.89 | 3.35 | 11.09 | 12.93 | 8.85 | 2.12 | 3.96 | 4.16 |
| BGIBMGA000903 | TCONS_00004700 | 0.38 | 0.20 | 0.01 | 1.00 | 0.00 | 0.06 | 0.14 | 0.00 |
| BGIBMGA000903 | TCONS_00004698 | 1.51 | 1.25 | 7.10 | 3.78 | 2.79 | 0.17 | 3.80 | 1.00 |
| BGIBMGA000903 | TCONS_00004693 | 3.98 | 0.28 | 3.96 | 5.91 | 6.08 | 4.50 | 4.61 | 5.10 |

**Table S4.** The levels of MG-specific genes with transmembrane transport function induced by BmCPV.

| Gene ID | Isoform ID | FB 0h | FB 3h | FB 24h | FB72h | MG 0h | MG 3h | MG 24h | MG 72h |
| --- | --- | --- | --- | --- | --- | --- | --- | --- | --- |
| BGIBMGA001299 | TCONS_00004032 | 0.42 | 0.00 | 0.10 | 0.02 | 4.39 | 1.14 | 1.45 | 2.23 |
| BGIBMGA001299 | TCONS_00004033 | 2.47 | 0.22 | 0.25 | 7.42 | 14.99 | 30.41 | 37.54 | 53.28 |
| BGIBMGA001299 | TCONS_00004031 | 6.08 | 0.53 | 0.03 | 11.12 | 31.28 | 44.74 | 50.39 | 67.91 |
| BGIBMGA014248 | TCONS_00054116 | 3.73 | 2.27 | 1.42 | 4.19 | 6.21 | 9.92 | 11.76 | 21.66 |
| BGIBMGA014248 | TCONS_00054113 | 5.50 | 2.77 | 1.81 | 4.13 | 9.24 | 6.50 | 13.22 | 11.18 |
| BGIBMGA014248 | TCONS_00054115 | 12.51 | 5.72 | 2.11 | 8.61 | 22.81 | 41.99 | 40.10 | 52.13 |
| BGIBMGA014434 | TCONS_00056498 | 0.63 | 0.00 | 0.02 | 1.56 | 4.52 | 5.51 | 6.36 | 6.37 |
| BGIBMGA014434 | TCONS_00056497 | 3.44 | 0.05 | 0.07 | 7.87 | 22.92 | 37.16 | 33.88 | 41.51 |
| BGIBMGA014434 | TCONS_00056499 | 1.52 | 0.07 | 0.07 | 2.20 | 6.88 | 12.03 | 15.42 | 13.72 |
| BGIBMGA004804 | TCONS_00018472 | 2.22 | 0.76 | 0.24 | 0.70 | 3.95 | 5.29 | 4.28 | 3.57 |
| BGIBMGA004804 | TCONS_00018473 | 11.56 | 2.22 | 1.60 | 8.09 | 29.98 | 42.53 | 29.64 | 27.17 |
| BGIBMGA009201 | TCONS_00035440 | 0.01 | 0.01 | 0.01 | 4.16 | 10.71 | 6.47 | 0.01 | 4.05 |
| BGIBMGA009201 | TCONS_00035439 | 2.47 | 0.03 | 0.34 | 6.72 | 12.10 | 30.25 | 31.89 | 25.07 |
| BGIBMGA009201 | TCONS_00035438 | 2.05 | 0.03 | 0.07 | 4.15 | 11.62 | 18.48 | 19.42 | 19.73 |
| BGIBMGA009201 | TCONS_00035437 | 4.35 | 0.06 | 0.14 | 11.40 | 34.00 | 50.30 | 39.12 | 46.74 |
| BGIBMGA009201 | TCONS_00035441 | 10.91 | 0.37 | 0.50 | 4.66 | 49.62 | 46.99 | 49.93 | 37.95 |
| BGIBMGA012068 | TCONS_00046109 | 1.00 | 0.00 | 0.04 | 0.79 | 4.20 | 7.52 | 5.91 | 5.33 |
| BGIBMGA012068 | TCONS_00046110 | 0.27 | 0.00 | 0.00 | 0.49 | 2.22 | 3.92 | 2.54 | 4.14 |
| BGIBMGA012068 | TCONS_00046107 | 0.19 | 0.00 | 0.01 | 0.99 | 2.04 | 4.30 | 4.33 | 3.86 |
| BGIBMGA012068 | TCONS_00046106 | 0.77 | 0.00 | 0.06 | 2.40 | 7.00 | 8.92 | 8.96 | 11.10 |
| BGIBMGA012068 | TCONS_00046112 | 0.00 | 0.03 | 0.01 | 0.84 | 2.03 | 3.04 | 2.05 | 6.06 |
| BGIBMGA012068 | TCONS_00046108 | 0.82 | 0.06 | 0.04 | 0.90 | 3.05 | 4.73 | 2.50 | 3.82 |

**Table S5.** The levels of MG-specific genes related to immunity induced by BmCPV.

| Gene ID | Isoform ID | FB 0h | FB 3h | FB 24h | FB72h | MG 0h | MG 3h | MG 24h | MG 72h |
| --- | --- | --- | --- | --- | --- | --- | --- | --- | --- |
| BGIBMGA008167 | TCONS_00031554 | 3.95 | 0.03 | 0.03 | 7.76 | 23.70 | 40.35 | 33.50 | 30.28 |
| BGIBMGA008167 | TCONS_00031553 | 2.19 | 0.06 | 0.04 | 8.40 | 18.78 | 35.28 | 29.41 | 26.62 |
| BGIBMGA009461 | TCONS_00036649 | 2.63 | 0.31 | 0.56 | 6.35 | 13.76 | 20.01 | 21.36 | 21.73 |
| BGIBMGA009461 | TCONS_00036648 | 1.39 | 0.53 | 0.34 | 2.79 | 7.25 | 8.32 | 8.32 | 9.36 |
| BGIBMGA012452 | TCONS_00047968 | 0.00 | 0.00 | 0.00 | 8.45 | 72.50 | 132.06 | 134.15 | 176.49 |
| BGIBMGA012452 | TCONS_00047969 | 0.00 | 0.02 | 0.00 | 0.00 | 4.48 | 13.19 | 6.57 | 18.96 |
| BGIBMGA012452 | TCONS_00047963 | 20.57 | 0.63 | 0.32 | 27.56 | 37.19 | 31.61 | 48.92 | 28.26 |
| BGIBMGA012452 | TCONS_00047962 | 1.27 | 1.48 | 0.91 | 0.80 | 2.81 | 3.74 | 4.49 | 4.45 |
| BGIBMGA013275 | TCONS_00050356 | 15.28 | 0.00 | 0.03 | 7.69 | 99.31 | 146.76 | 93.65 | 200.88 |
| BGIBMGA013275 | TCONS_00050358 | 1.15 | 0.06 | 0.12 | 8.71 | 12.20 | 36.06 | 10.85 | 19.07 |
| BGIBMGA013275 | TCONS_00050353 | 38.35 | 0.14 | 0.07 | 85.37 | 245.38 | 310.59 | 390.80 | 488.69 |
| BGIBMGA007987 | TCONS_00031401 | 101.08 | 10.29 | 11.00 | 194.41 | 547.89 | 701.90 | 880.72 | 1127.77 |
| BGIBMGA007987 | TCONS_00031402 | 55.47 | 19.84 | 10.80 | 73.77 | 164.58 | 271.94 | 319.10 | 316.27 |
| BGIBMGA007987 | TCONS_00031403 | 99.64 | 30.89 | 19.13 | 156.41 | 311.88 | 477.96 | 595.34 | 574.41 |

**Table S6. Primers summary.**

| Name | Forward primer | Reverse primer |
| --- | --- | --- |
| BGI007453 | CACCAAAGCCGTTGAGAAAG | TCCGAAGAGCGGGACCA |
| BGI008203 | GTGGCTATTGTATTCTTTCGTCG | GTTTGATGTTGTGCTGCTTGAG |
| BGI009576 | CTGCGTATGGTTGGTATGTGG | GCTGCGATGCTTGCTCTAA |
| BGI014087 | GCGAGCACTTCACATTTCAC | CCGCCGTCAACTCACAAA |
| BGI000903 | GTTTGCTAACTGCCCACCC | CTGCCAATGCTGTCGCTAT |
| BGI001299 | TCTCATCTTCCTGGTCTTCGC | TCGTTCTCAACTGGCATTACTACTC |
| BGI014248 | GCTCAACTCGGCACAATCAT | CCCCAAGTACAAGCCACAGA |
| BGI014434 | TGGGACCCTGGTGGTTCAT | TTCTTGCGGTTCGTTTCTCA |
| BGI004804 | AAACTTATTTCTCGTCGTGGTG | CGCAGTGCCCTTTATCCTC |
| BGI009201 | CCTTATCGTCTCATACTACTGGCTAC | AGGGTCTGCTTCTACGGGTT |
| BGI012068 | ATGAATAGGAATGACGCTAATCTTG | ACCCTTCGGACTCGGAAATAC |
| BGI008167 | ATGGCGACTTGGATGCG | CCTTCGCTCGTGGTCAATAA |
| BGI009461 | CAGCCGTGGATGATGAGG | TGTAAAGCTATCTGGACGTTGG |
| BGI012452 | CCTTGTGGGAGGTGAATGG | GTTTGCCCGTAATACCGATG |
| BGI013275 | TCTTTGGCGTCGCACCC | ATCCCTGATAATCCTCGTTTCTG |
| BGI007987 | TATGCTTCTGGCTGGGTTCA | TGGTTCCATCCCGCTCCT |
| sw22934 | TTCGTACTGGCTCTTCTCGT | CAAAGTTGATAGCAATTCCCT |
